# PIM kinase inhibition: co-targeted therapeutic approaches in prostate cancer

**DOI:** 10.1038/s41392-020-0109-y

**Published:** 2020-01-31

**Authors:** Sabina Luszczak, Christopher Kumar, Vignesh Krishna Sathyadevan, Benjamin S. Simpson, Kathy A. Gately, Hayley C. Whitaker, Susan Heavey

**Affiliations:** 1 Molecular Diagnostics and Therapeutics Group, University College London, London, UK; 2 Trinity Translational Medicine Institute, St. James’s Hospital Dublin, Dublin 8, Dublin, Ireland

**Keywords:** Molecular medicine, Germ cell tumours

## Abstract

PIM kinases have been shown to play a role in prostate cancer development and progression, as well as in some of the hallmarks of cancer, especially proliferation and apoptosis. Their upregulation in prostate cancer has been correlated with decreased patient overall survival and therapy resistance. Initial efforts to inhibit PIM with monotherapies have been hampered by compensatory upregulation of other pathways and drug toxicity, and as such, it has been suggested that co-targeting PIM with other treatment approaches may permit lower doses and be a more viable option in the clinic. Here, we present the rationale and basis for co-targeting PIM with inhibitors of PI3K/mTOR/AKT, JAK/STAT, MYC, stemness, and RNA Polymerase I transcription, along with other therapies, including androgen deprivation, radiotherapy, chemotherapy, and immunotherapy. Such combined approaches could potentially be used as neoadjuvant therapies, limiting the development of resistance to treatments or sensitizing cells to other therapeutics. To determine which drugs should be combined with PIM inhibitors for each patient, it will be key to develop companion diagnostics that predict response to each co-targeted option, hopefully providing a personalized medicine pathway for subsets of prostate cancer patients in the future.

## Introduction

Prostate cancer (PCa) is one of the most common cancers among men, with 1.1 million cases per year worldwide.^1^ As current treatments,^2,3^ including surgery, radiotherapy, chemotherapy, and hormone therapy, result in severe side effects,^4^ the development of new targeted therapies with lower toxicity could significantly improve patient quality of life and potentially extend life. Moreover, neoadjuvant therapeutics effective at reducing tumor volume could potentially allow better preservation of erectile function and urinary continence in radical prostatectomy patients.^2,5^

Tumorigenesis in PCa is often dependent on aberrations in one of the key signal transduction pathways, several of which interact with the PIM family.^6^ The PIM family (proviral integration site for Moloney murine leukemia virus) consists of three serine/threonine kinases, which are known to be overexpressed in PCa, as well as breast cancer and hematological malignancies, and are often correlated with decreased overall survival (OS), resistance to therapy and cancer cell proliferation.^7^ PIM proteins have been implicated in driving cell growth and survival, proliferation, and avoidance of apoptosis^7^ by interacting with other tumorigenic pathways, such as the PI3K (phosphoinositide 3-kinase)/mTOR (mammalian target of rapamycin)/AKT (protein kinase B) pathway,^8^ as well as by influencing oncogenes and tumor suppressor genes.^8^ Moreover, PIM upregulation can cause resistance to conventional chemotherapy,^9^ radiotherapy,^10^ PI3K inhibitors^11^, and other therapeutics.^12^ The activity of PIM is primarily regulated at the transcriptional and protein stabilization level and is mainly influenced by the JAK/STAT (Janus kinase/signal transducer and activator of transcription) pathway, NF-κB (nuclear factor kappa-B)^8^ and HSP90 (heat shock protein 90).^13,14^

This large repertoire of PIM signaling interactions and its implication in resistance to other treatment modalities provide a rationale for co-targeting PIM with other therapies in order to increase its efficacy.

## Role of PIM in prostate cancer

The PIM family is composed of three highly conserved serine/threonine kinases—PIM1, PIM2, and PIM3. PIM1 has been identified to have two isoforms (33 and 44 kDa), PIM2 has three isoforms (34, 37 and 40 kDa), and PIM3 has one isoform.^8^ Xie et al. suggested that functional differences existed between the long and short isoforms of PIM1, as the 44 kDa isoform (PIM1L) is mainly present on the plasma membrane. and the 33 kDa isoform (PIM1S) is mostly in the nucleus. PIM1L interacts with the SH3 (SRC homology 3) domain of the Etk tyrosine kinase, which has been shown to be one of the sources of resistance to chemotherapeutic drugs in PCa cell models.^15^

The oncogenic potential of the PIM family is perhaps best characterized within PCa, where extensive work has been carried out. Data are available that suggest a role for PIM1 in particular, with higher expression of PIM1 or PIM3 noted in PCa versus matched benign tissues in multiple cohorts.^16–20^ This increase in expression of PIM has prompted numerous studies investigating the role of the whole PIM family in the development and progression of PCa.^21^ Interestingly, the impact of PIM on patient prognosis is disputed, as some reports suggest that low PIM1 expression in prostate cancer can be linked to poor patient outcomes.^22^

PIM1 and PIM2 have been shown to play a role in PCa tumorigenesis, with PIM1 overexpression increasing the tumorigenicity of two PCa cell lines, LNCaP and DU145, both in vitro and in vivo,^23^ while PIM2 has been suggested to play a role in prostate tumorigenesis via phosphorylation of eIF4B (eukaryotic translation initiation factor 4E).^24^ It has been noted that this PIM-mediated initiation of prostate tumors is weak and that the kinase family may play a bigger role in the progression of the disease.^25^ PIM1, in particular, has been implicated in that process, notably contributing to invasion, migration and metastasis in vivo.^26,27^ In transgenic mouse models, PIM1 and PIM2 have also been shown to be overexpressed in prostate tumors that possess inflammatory features and markers of stemness, underpinning their relevance to aggressive, drug-resistant, advanced disease.^17^ PIM2 overexpression is perhaps more clinically important in various hematological cancers, but it has also been shown to be linked to increased proliferation and reduced apoptosis in prostate cancer.^28^ Other hallmarks of cancer that are to some extent influenced by PIM expression include angiogenesis,^29^ migration, and invasion,^30–32^ evasion of the immune response^33^ and modulation of energy metabolism.^34,35^ Unfortunately, most data regarding PIM expression and its clinical impact have been obtained from mouse models and refer mainly to mRNA expression, which may not be an accurate representation of translated kinase levels. This issue could be rectified by employing carefully vetted antibodies to be used in immunohistochemistry analysis of human prostate cancer samples, which would provide researchers with a more accurate representation of human malignancy.^36^

Perhaps PIM’s most significant role is in cooperating with other oncogenes, leading to pronounced aggressive phenotypes. One key oncogene that is regulated by PIM is c-MYC (avian myelocytomatosis viral oncogene homolog). Cells expressing both c-MYC and PIM showed increased tumor progression, leading to an advanced cancer phenotype, and silencing of PIM1 resulted in c-MYC-related tumor inactivation, suggesting that PIM1 may be required to maintain c-MYC-driven aggressive PCa.^37,38^ Other downstream PIM targets include p27 (cyclin-dependent kinase inhibitor 1B)^39,40^ and BAD (Bcl2-associated death promoter).^8,41^

PIM kinases have also been shown to contribute to the stabilization of the tumor suppressor NKX3.1 by protecting it from proteasome-mediated degradation. Expression of NKX3.1 has been suggested to limit the tumorigenesis of PCa cells and suppress tumor growth. Paradoxically, PIM kinases, despite functioning mainly as oncogenes, protect this prostate-specific tumor suppressor and its anti-tumor activity. Moreover, PIM inhibition reduced NKX3.1 levels.^42^ This relationship emphasizes the complexity of signaling pathways in tumors and suggests that PIM therapy may have a detrimental effect on some tumor suppressors.

## PIM inhibition as a monotherapy

To date, most efforts to inhibit PIM in cancer treatment have focused on a monotherapeutic approach, generally using ATP (adenosine triphosphate)-competitive drugs that target the kinase action of the protein, preventing it from phosphorylating its downstream effectors, either through quinones or other classes of small molecule inhibitors^43–45^ (Table 1).

**Table 1:** Clinical trials investigating the use of PIM inhibitors in cancer.

Trial	Target(s)	Drug	Drug class	Cancer type(s)	Phase	Status
NCT00848601	pan-PIM	SGI-1776	Imidazopyridazine-based inhibitor	Refractory Prostate Cancer and Relapsed/Refractory Non Hodgkin's Lymphoma	I	Terminated
NCT01239108	pan-PIM	SGI-1776	Imidazopyridazine-based inhibitor	Relapsed/Refractory leukemias	I	Withdrawn
NCT01489722	pan-PIM	AZD1208	Thiazolidinedione-based inhibitor	Acute myelogenous leukemia	I	Terminated
NCT01588548	pan-PIM	AZD1208	Thiazolidinedione-based inhibitor	Advanced solid tumors and malignant lymphoma	I	Completed
NCT02370706	pan-PIM	PIM447	Fluoropicolinamide-based inhibitor	Myelofibrosis	I	Active, not recruiting
NCT02078609	Pan-PIM	PIM447	Fluoropicolinamide-based inhibitor	Acute myeloid leukemia or high risk myelodysplastic syndrome	I	Completed
NCT03715504	Pan-PIM (greater efficacy for PIM-1)	TP-3654	Pyrazololpyrimidine-based inhibitor	Advanced solid tumors	I	Recruiting
NCT03008187	Pan-PIM & FLT3	SEL24	Benzodiazole-based inhibitor	Acute myeloid leukemia	I/II	Recruiting

Limited clinical studies have been carried out investigating PIM inhibition in prostate cancer to date. Targeted inhibition of PIM is more developed with regard to hematological malignancies than for solid tumors

AZD1208 is one of the most developed small molecule PIM inhibitors. It is a selective ATP-competitive pan-PIM kinase inhibitor that controls the expression and phosphorylation of downstream PIM effectors such as STAT3 or mTOR.^46^ In PCa, AZD1208 inhibits cell growth, induces apoptosis, inhibits motility and alters cellular morphology, as well as acting as a radiation sensitizer.^47^ A phase I dose-escalation study carried out in 35 solid tumor patients showed that AZD1208 elicited no functional response, even though the PIM kinase was successfully inhibited, and it was suggested that the clinical efficacy of the drug might increase in combination with other targeting agents.^48^ Overall, 66 out of 67 patients reported adverse effects posttreatment, including gastrointestinal disorders (nausea and diarrhea) and fatigue. Seventy-five percent of acute myeloid leukemia (AML) patients and 45.7% of solid cancer patients experienced grade ≥3 adverse effects according to the CTCAE (Common Terminology Criteria for Adverse Effects), including febrile pneumonia, neutropenia, hypotension, and maculopapular rash. Because of the high toxicity, 4 solid tumor patients discontinued their treatment.^48^

TP-3654 (SGI-9481) is a second-generation small molecule pan-PIM inhibitor with improved potency and decreased cardiotoxicity compared to previous PIM inhibitors. Notably, this drug selectively targets PIM1, with a Ki (inhibition constant) of 5 nm for PIM1, 239 nM for PIM2 and 42 nM for PIM3. Moreover, it reduced phospho-BAD expression, exhibited no treatment toxicity and reduced tumor volume.^49,50^ This pattern of selectivity is often replicated by other PIM inhibitors, as many of them inhibit PIM2 much less efficiently than other PIM kinases.^51^

A series of quinone analogs have been shown to offer selective inhibition of PIM, leading to attenuation of growth in the PCa cell line DU145.^43^ The PIM selective inhibitor DHPCC-9 impairs the antiapoptotic effects of PIM1 and inhibits the intracellular phosphorylation of PIM substrates, including BAD, leading to reduced invasion and migration in PCa models.^30^ Another small molecule PIM inhibitor, SMI-4a, has been well studied in hematological malignancies, where it has been shown to induce apoptosis and has been recommended for study in PCa models.^52^ CX-6258 is an oxindole-based pan-PIM inhibitor that also shows some effect against FLT-3 (Fms-like tyrosine kinase 3)^53^ and is under preclinical testing, with some evidence to suggest a role in PCa treatment, especially in combination with other therapeutics such as CX-6461 (refs. ^32,54^). In AML models, it has been shown to be greatly selective, be well tolerated by mouse carriers, have an anti-proliferative effect, block phosphorylation of BAD and 4E-BP1 (eukaryotic translation initiation factor 4E binding protein 1), and potentiate the antiproliferative effect of chemotherapeutic agents, such as doxorubicin and paclitaxel.^55^ PIM447 is a selective small molecule pan-PIM inhibitor that has not yet been investigated in PCa; however, the results in large B-cell lymphoma suggest that PIM inhibition by this drug leads to inactivation of the mTORC1 signaling complex, followed by the inactivation of cap-dependent protein translation, thereby leading to apoptosis and cell death.^56^ This suggests that the known interactions between PIM and PI3K signaling could offer broad signaling effects in response to PIM inhibition as a monotherapy.

Currently, one of the challenges of PIM-targeted therapy is the toxicity of some inhibitors. A clinical trial investigating the efficacy of SGI-1776, a pan-PIM and FLT-3 inhibitor, in prostate cancer and non-Hodgkin lymphoma was terminated early due to its cardiotoxicity. It has been suggested that prolongation of the cardiac QT has been a result of inhibition of the cardiac potassium channel human ether-à-go-go-related gene (hERG).^50^ More novel inhibitors, such as TP-3654 (SGI-9481), have been demonstrated to be better tolerated; however, their effect on hERG and FLT-3 should be evaluated prior to clinical trials to address the issue of potential treatment toxicity.

## PIM inhibition within the prostate cancer clinical pathway

**Fig. 1:**
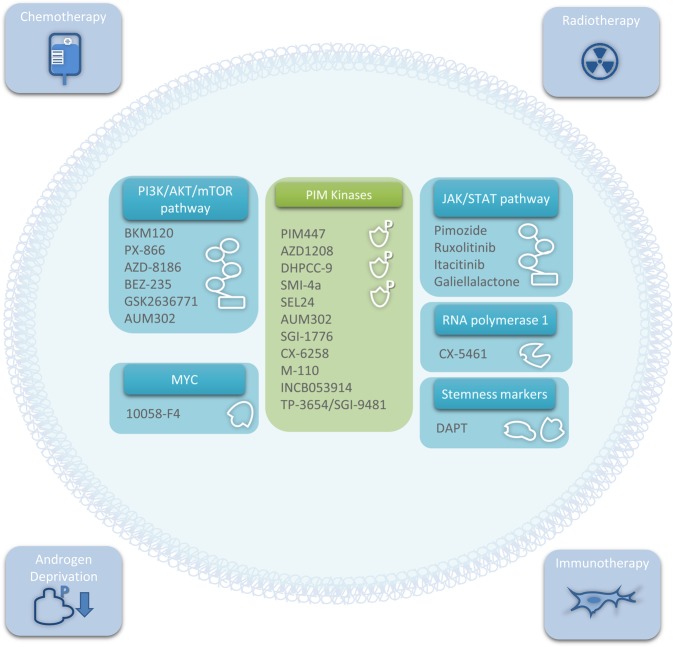
Categories of therapeutics that could be used in combination with PIM inhibition in prostate cancer. Outer boxes: therapies that are well developed for prostate cancer but that could benefit from the PIM co-targeted inhibition approach. Inner boxes: related targets and specific drugs that are currently under development. Abbreviations: AKT, protein kinase B; EGFR, epidermal growth factor receptor; HER2, human epidermal growth factor receptor 2; mTOR, mammalian target of rapamycin; PI3K, phosphoinositide 3-kinase.

Currently, the PCa clinical pathway includes, depending on the risk stratification, active surveillance, surgical prostatectomy, radiotherapy (external beam intensity-modulated and brachytherapy),^2^ hormone therapy,^57^ chemotherapy and radium-223 (ref. ^3^) (Fig. 1). However, most of these therapies can result in severe side effects and decrease patient quality of life.^2,58^ Successful PIM-targeted therapy could be used as a neoadjuvant drug prior to surgery to shrink the tumor and enable more patients to undergo nerve-sparing radical prostatectomy and therefore improve functional outcomes.^5^ Moreover, it has been demonstrated that PIM inhibitors can enhance the efficacy of existing PCa treatments, which will be discussed further, including radiotherapy,^10^ chemotherapy,^9^ and androgen deprivation.^59^ PIM co-targeting could also reduce patient mortality by potentiating the antimetastatic effects of other treatments, including strategies targeting the PI3K pathway^60,61^ or JAK/STAT pathway^62,63^ or anti-androgen therapy.^64^

## PIM and the PI3K pathway

The PI3K/AKT/mTOR pathway has been shown to contribute to the development of all hallmarks of cancer and is frequently disrupted in cancer.^65^ Three generations of drugs targeting this pathway have been under widespread clinical development for multiple cancers.^66^ However, many have exhibited issues with toxicity or resistance due to compensatory mechanisms and feedback loops with closely related signaling pathways, and co-targeted inhibition approaches have been gaining popularity in preclinical studies.^67–70^ Thus, PIM and PI3K co-targeting may be a viable approach owing to the overlap between those pathways, the proven PIM-induced resistance to PI3K inhibition^35^ and the reported successful synergism that results when both are used.^71^ Synergy between inhibitors could allow the use of lower therapeutic doses to achieve the same clinical effect, thus potentially reducing treatment toxicity and improving patient quality of life.

**Fig. 2:**
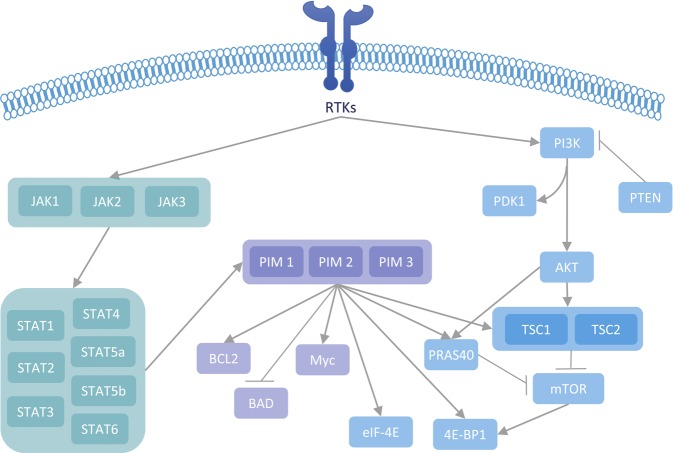
Key interactions between PIM kinases and targetable signaling pathways. In prostate cancer, the key targetable pathways that interact closely with PIM signaling are the JAK/STAT and PI3K pathways. Abbreviations: 4E-BP1, eukaryotic translation initiation factor 4E binding protein 1; AKT, protein kinase B; BAD, Bcl2-associated death promoter; BCL2, B-cell CLL/lymphoma 2; eIF-4E, eukaryotic translation initiation factor 4E; JAK, Janus kinase; mTOR, mammalian target of rapamycin; MYC, avian myelocytomatosis viral oncogene homolog; PDK1, pyruvate dehydrogenase kinase 1; PI3K, phosphoinositide 3-kinase; PIM, proviral integration site for Moloney murine leukemia virus; PRAS40, proline-rich Akt substrate, 40 KDa; PTEN, phosphatase and tensin homolog; RTK, receptor tyrosine kinase; STAT, signal transducer and activator of transcription; TSC, tuberous sclerosis complex.

The PIM and PI3K/AKT/mTOR pathways overlap and influence each other in multiple ways (Fig. 2). PIM mimics the cellular functions of AKT, leading to similar effects on molecules influencing the cell cycle, survival, apoptosis and growth.^8^ This includes the phosphorylation of cell cycle and proliferation mediators, such as p21wafl (cyclin-dependent kinase inhibitor 1A), p27kip (cyclin-dependent kinase inhibitor 1B), and Mdm2 (mouse double minute 2), as well as pro-apoptotic members of the Bcl-2 family (B-cell lymphoma gene), such as BAD. Moreover, both PIM and AKT block the caspase-induced apoptotic processes by disrupting caspase 3 and caspase 9 (ref. ^8^). Another point of convergence of the PIM and PI3K pathways is mTOR, a molecule downstream of PI3K that is responsible for cellular processes, including cell survival and protein synthesis, and influences the pathogenesis of disorders such as cancer and type 2 diabetes.^72,73^ Both PIM and AKT negatively regulate mTOR by phosphorylation of PRAS40 (proline-rich Akt substrate of 40 kDa)^74^ and TSC2 (tuberous sclerosis complex 2),^75^ the latter of which occurs by both directly and indirectly decreasing AMPK (5' adenosine monophosphate-activated protein kinase) activation.^8^ Moreover, mTOR and PIM can phosphorylate 4E-BP1 to allow for protein translation and MYC signaling.^76^ Another link between the pathways is the phosphorylation of c-MYC, which prevents apoptosis and allows the oncogene to drive tumorigenesis.^7,8,77,78^

A number of clinical trials have been conducted to investigate the effects of PI3K inhibition in solid tumors, including PCa. The drugs under review in phase I or II trials include BKM120 (NCT01385293, NCT01695473, and NCT02035124), AZD-8186 (NCT01884285 and NCT03218826), GSK2636771 (NCT02215096), PX-866 (NCT01331083) and BEZ-235 (NCT01717898 and NCT01634061). However, many PI3K pathway inhibitors failed to show substantial clinical results as monotherapies, partially owing to the development of toxicity, including autoimmune toxicities, hypertension, neuropsychiatric effects, diarrhea, respiratory complications and susceptibility to infection, and treatment resistance.^79,80^

Resistance mechanisms have been linked to PI3K’s ability to downregulate negative feedback loops once inhibited, as well as to its crosstalk with other signaling pathways including the MAPK (mitogen-activated protein kinase) pathway and the androgen receptor (AR) pathway.^79^ The PIM family has also been implicated in PI3K pathway inhibitor failure, which mainly stems from common substrates affected by both pathways. A suggested mechanism for resistance involves PIM regulation of mTORC1 (ref. ^35^) and protection of its function despite upstream PI3K inhibition.^11^ FLT3-ITD (internal tandem duplication) has been shown to upregulate STAT5 and subsequently cause PIM overexpression, as well as activate both the PI3K/Akt/mTOR and MAPK pathways, which can all stimulate mTOR signaling.^11^

Efforts have begun to co-target PIM and the PI3K pathway, which has exhibited promising synergistic effects, with the combined therapy suppressing cell growth^35,81^ and viability and increasing apoptosis^11^ in comparison with monotherapies in PCa,^71^ as well as in hematological malignancies.^11,82^

Another study on human PCa cells^35^ supported these findings, showing that combined therapy resulted in lower cell growth and survival than the monotherapies in vitro, as well as higher tumor shrinkage and reduced proliferation in mouse models. The study also presented evidence in favor of the relationship between PIM, mTORC1, NRF2 (nuclear factor erythroid 2-related factor, a molecule involved in response to oxidant stress),^83^ and the resultant mechanisms of PI3K inhibitor resistance to apoptosis. Co-targeting PIM and PI3K was more effective than monotherapy in decreasing cell proliferation via accumulation of reactive oxygen species (ROS), a consequence of NRF2 inhibition. As PIM1 increases the synthesis of glutathione, the suppression of both glutathione and PI3K caused a significant increase in ROS accumulation, leading to lower tumor volume; this relationship between PIM1 and glutathione expression could further explain the complex role the PIM family plays in PI3K resistance.^83^

A novel drug targeting PIM/PI3K/mTOR, AUM302 (IBL-302), has recently been reported in neuroblastoma.^84^ It has been shown to be very specific, more bioavailable than earlier drugs of the same pipeline and effective in decreasing levels of downstream targets of PIM/PI3K/mTOR, including phosphorylated AKT and N-Myc. Moreover, AUM302 increased the differentiation of patient-derived xenografts and cancer cell culture models, reduced cell viability and tumor growth, and potentiated the effect of doxorubicin, etoposide, and cisplatin, with the possibility of reducing the dosage of cisplatin by 50% while retaining its therapeutic effect. In comparison to dactolisib (BEZ-235), a PI3K/mTOR inhibitor, AUM302 was superior in its ability to decrease cell viability.^84^

These initial studies suggest that PIM/PI3K co-targeting has potential clinical benefit, although further in vitro and in vivo experiments should be carried out using different inhibitors prior to PIM-PI3K co-targeted clinical trials.

## PIM and the JAK/STAT pathway

PIM is primarily activated by the JAK/STAT signaling pathway, which results in phosphorylation of its downstream targets^17^ (Fig. 2). In response to stimuli such as interleukins, downstream STAT dimers translocate to the nucleus,^8^ where STAT3 and STAT5 have been shown to bind to PIM1 promoters, in particular,^8^ promoting aggressive, invasive PCa.^85,86^ It has been suggested that STAT3 does this by aiding myeloid-derived suppressor cells (MDSCs) in their effort to suppress antitumor activity.^87^ STAT3 has been shown to be upregulated in as many as 82% of PCa tumors compared to surrounding benign tissue.^88^ Moreover, PIM1 upregulation by JAK/STAT activates a negative feedback loop, in which it activates suppressors of cytokine signaling (SOCSs), which prevent JAK/STAT signaling transduction.^8,89^

Given the success of vertical co-targeting approaches in the PI3K pathway, where PI3K and mTOR are frequently co-targeted using dual inhibition strategies,^90^ we should consider the option of vertically co-targeting the PIM pathway with upstream proteins such as STAT3 and STAT5, as opposed to purely horizontal co-targeting approaches with other signaling pathways. Targeting STAT3/STAT5 in PCa has previously been identified as a promising avenue for investigation, with the psychotropic inhibitor pimozide indirectly inhibiting STAT5 and leading to an apoptotic response^91^ and STAT3 inhibition offering promise in PCa treatment.^92,93^ The STAT3-targeted inhibitor galiellalactone has been shown to be successful in preventing the generation of MDSCs and thus suppressing the immune response against PCa cells.^87^ Inhibition of PIM by M-110 and SGI-1776 can indirectly decrease levels of phosphorylated STAT3, but not STAT5, in cell models, which contributed to downregulation of PIM3 (ref. ^94^). Early work suggests efficacy of the co-targeting strategy in myelofibrosis, leading to a clinical trial combining ruxolitinib, a JAK1/JAK2 inhibitor, with PIM447, a pan-PIM inhibitor^95^ (NCT02370706), and another trial combining JAK1 inhibition (itacitinib) with a preclinical PIM inhibitor (INCB053914) in hematological malignancies.^82^ Contrary to these results, another study found that inactivation of STAT3 or IL-6 (interleukin-6) could lead to quicker PCa progression, as it would prevent STAT3 stimulation of p14 (ARF) and its other tumor suppressor targets.^96^ This evidence would argue that targeting STAT would not be beneficial for the patients, and high levels of STAT3 should be treated as a biomarker and not a therapeutic target.^96^ We propose that a co-targeted approach of inhibiting PIM along with STAT3/5 could potentially offer improved anticancer effects, particularly in the setting of advanced PCa; however, such a strategy should be approached with caution and researched in depth to verify whether it could be a viable treatment option.

## PIM and MYC

PIM1 and MYC are often coexpressed in PCa and can both influence its development and progress. It has been suggested that PIM itself is only weakly tumorigenic but that it has the ability to potentiate the effect of other oncogenes such as MYC^37^ by increasing its transcriptional activity or stability.^38^ Interestingly, it promotes the oncogenic properties of MYC signaling while suppressing its ability to promote apoptosis.^97^ PIM acts on a large proportion of MYC target genes (41%), including connective tissue growth factor (CTGF) and CD24 (ref. ^23^). Coexpression of PIM and MYC has been shown to result in increased proliferation and apoptosis, the latter possibly due to increased cell turnover.^37^ Moreover, PIM and MYC coexpression can be correlated with a higher Gleason grade of PCa.^37^ MYC promotes prostate tumorigenesis by affecting ribosomal DNA and RNA, activation of pro-tumorigenic genes such as EZH2 (enhancer of zeste homolog 2) and downregulation of tumor suppressor genes such as NKX3.1 (homeobox protein Nkx-3.1).^97^

It has been demonstrated that inhibition of c-MYC using 10058-F4 suppresses the colony formation of PIM1-expressing PCa cell models (LNCaP and DU145 cells), as well as the expression of PIM1 itself at the protein level.^23^ Interestingly, Wang et al suggested that the effects of PIM1 knockdown on MYC expression varied between different cell lines (DU145 and MPT).^38^ Moreover, PIM inhibitors such as AZD1208 were effective at downregulating MYC activity, inhibiting the growth of MYC-driven PCa, increasing apoptosis and reducing proliferation (BrdU index).^47^ Because of the synergistic effect of PIM and MYC, a co-targeting approach could enhance the effect of the treatment, or alternatively, PIM inhibition could be used to indirectly target the expression of c-Myc.^23^ The relationship between PIM and MYC is one of the examples of synthetic lethality and how it can be used to maximize the efficacy of existing drugs via inhibition of proteins related to the effector of interest.^98^ Other targets that exhibit synthetic lethality with PIM could be identified by genetic screening, which would uncover the epigenetic changes driving tumorigenesis in prostate cancer.

## PIM and stemness

PIM1 has been shown to play a role in the development of stemness phenotypes. In mouse embryonic and rat and ovine mesenchymal stem cells, PIM1 is upregulated, which may lead to self-renewal, increased proliferation and survival.^99–101^ Human cardiac stem/progenitor cells with transduced PIM1 exhibited lower senescence, longer telomeres, and higher telomerase activity, proliferation and cell survival than control cells.^102,103^ Moreover, PIM1 has been found to act on putative stem cell markers, including breast cancer resistance protein (BCRP)/adenosine triphosphate-binding cassette subfamily G member 2 (ABCG2), leading to its membrane translocation and development of resistance to chemotherapeutics.^59,104^ BCRP/ABCG2 has also been shown to guard prostate stem-like cells from the negative effects of androgen deprivation, hypoxia, and chemotherapeutic drugs, which encourages the development of PCa.^104^ As PIM1 may stimulate a stem-like phenotype within prostate cancer, targeting it could be effective in preventing progression in the early stages of the disease.

PIM kinases have been implicated in the Notch (neurogenic locus Notch homolog protein) embryonic pathway, which plays a role in the development of various cancers, including prostate and hematological malignancies,^105^ by inducing epithelial-to-mesenchymal transition and promoting resistance to chemotherapy and survival.^106,107^ PIM kinases have been shown to phosphorylate and stimulate the activity of Notch1, and activated Notch1 can upregulate the expression of PIM.^107^ Moreover, PIM kinases and Notch share some downstream targets, including MYC, p21, and NF-κB.^108^ It has been shown that inhibition of PIM (via the drug DHPCC-9) or Notch (via the drug DAPT) alone led to a reduction of tumor volume in PCa cell models; however, a co-targeting treatment approach proved more effective than any of the monotherapies and could be of interest for patients with aberrations in the PIM or Notch signaling pathways.^107^

## PIM and RNA polymerase I transcription

MYC has been shown to drive cell growth by upregulating ribosomal RNA synthesis. As mentioned previously, PIM is often coexpressed with MYC and can stimulate its transcriptional activity and stability, as well as act on other downstream targets, such as 4E-BP1, to contribute to PCa tumorigenesis and growth. Inhibition of RNA polymerase I, crucial for ribosome biogenesis, together with PIM kinases could synergistically block MYC signaling and thus produce better functional effects than targeting either alone. A study on PCa cell models has shown their sensitivity to treatment with inhibitors of RNA polymerase I and PIM (CX-5461 and CX-6258, respectively), with both drugs suppressing colony formation and causing cell cycle arrest.^32^ The effects of the treatments were potentiated with the combined therapies compared to those seen with either agent alone. Moreover, co-treatment with CX-5461 and CX-6258 in vivo reduced the tumor growth and proliferation and increased the apoptosis of patient-derived xenografts from castration-resistant PCa patients, which suggests that this combined approach could also be effective in patients with advanced diseases resistant to most current therapies.^32^ Similarly, the efficacy of CX-5461 together with CX-6258 in prostate ex vivo patient-derived xenografts has been demonstrated by another study, as the drugs, alone or in combination, were effective in all four types of diverse tumors; these results contrast with those achieved with other therapeutics, including the PARP (poly(ADP-ribose) polymerase) inhibitor talazoparib and the CDK4-6 (cyclin-dependent kinase) inhibitors ribociclib or cisplatin, which were effective against one or none of the samples.^54^ Moreover, the co-targeting approach upregulated the DNA damage response and downregulated downstream signaling from PIM and mTORC1 (ref. ^54^).

## PIM and androgen deprivation therapy (ADT)

PIM1 is known to phosphorylate AR, which plays a key role in PCa progression.^21,109^ Both PIM1S and PIM1L interact with AR, and differential phosphorylation of AR at multiple sites leads to modulation of stability and transcriptional activity.^110^ PIM-mediated phosphorylation of the receptor was observed in both the presence and absence of the hormone; this process was decreased upon treatment with PIM inhibitors.^109^ PIM is linked to castration-resistant PCa (CRPC) through pathways associated with c-MET (tyrosine-protein kinase Met), MYC and Oct4 (octamer-binding transcription factor 4), pathways believed to act as compensatory mechanisms in androgen deprivation therapy (ADT).^59^

PIM1 expression is maintained during androgen deprivation therapy in mice, implying that it may be important for maintenance of proliferation in this setting.^111^ AR levels do not appear to affect PIM1, which would suggest a promising approach for co-targeting, as the two are independently expressed. Investigations combining PIM inhibition and ADT are needed to assess whether the inhibition of PIM could overcome resistance to ADT.

## PIM and EGFR

EGFR (epidermal growth factor receptor) overexpression is commonly correlated with the development of many cancers, including prostate cancer.^112^ PIM1 expression is correlated with EGFR expression in head and neck cancer, in which anti-EGFR treatment (cetuximab and gefitinib) prevented the EGFR-stimulated translocation of PIM1 (ref. ^113^). In breast cancer, PIM1 has been implicated in driving resistance to HER2 (human epidermal growth factor receptor 2) via inactivation of BAD, thereby allowing cells to escape the effect of the drugs.^114^

In prostate cancer cell models, the PIM inhibitors M-110 and SGI-1776 successfully upregulated the expression of MIG6 (mitogen-inducible gene 6 protein), which prevents EGFR signaling. M-110 has also been shown to prevent EGF-stimulated activation and reduce the expression of EGFR, leading to lower ERK (extracellular signal-regulated kinase) pathway activity. Moreover, co-targeting PIM and EGFR using M-110 or SGI-1776 with gefitinib showed a synergistic effect on cell proliferation.^112^ This combination approach has also been shown in non-small-cell lung cancer (NSCLC), where AZD-1208 and osimertinib (an EGFR inhibitor) elicited synergistic effects with respect to cell viability and phosphorylation of STAT3 (ref. ^115^), which, if replicated in PCa, could prevent STAT3-driven promotion of aggressive prostate cancer features.^86^

## PIM and radiotherapy

PCa radiotherapy resistance is often linked to and may be attributed to aberrations in the expression of various signaling pathways and genes involved in cell growth and cell death, including downregulation of DOC2/DAB2 (double C2 domain; DAB adaptor protein 2) and P53 (tumor protein P53) and upregulation of MDM2 (ref. ^116^), epidermal growth factor (EGF), BCL-2 (ref. ^117^), and the PI3K/Akt/mTOR pathway.^118–120^ Other factors contributing to radioresistance may include cancer stem cells^121,122^ and the tumor microenvironment.^120^ Recently, PIM has also been implicated in contributing to tumor radioresistance.^10,123–125^

Upregulation of PIM has been noted following exposure to radiation, which leads to PIM translocation into the nucleus, phosphorylation of PRAS40 and formation of a complex of phospho-PRAS40, 14-3-3 protein and phospho-FOXO3a (forkhead box O3), which accumulates in the cytoplasm. This accumulation could lead to resistance to therapy and low levels of proapoptotic genes.^125^ PIM inhibition disallows phosphorylation of PRAS40 and results in increased nuclear FOXO3a, which increases the level of pro-apoptotic proteins (e.g., Fas and Fas ligand) and reduces radioresistance.^10^ Increased expression of PIM1 correlates with reduced efficacy of radiotherapy and increased expression of EGFR, which has been shown to act on PIM1 and stimulate its nuclear translocation.^113^

A study by Kirschner et al. investigated the relationship between radiation and PIM expression as well as the effect of a PIM inhibitor (AZD1208) on PCa radioresistance.^47^ They showed that radiation and hypoxia increased the expression of PIM1, which in turn led to increased efficacy of the drug. This approach was also effective at reducing tumor recurrence. Similar results, showing an encouraging synergistic effect of combining PIM inhibition and radiotherapy, have been reported for pancreatic cancer.^126^

## PIM and chemotherapy

PCa studies have suggested a potential role for PIM1 in the development of acquired resistance to chemotherapeutic agents,^9,15^ with further data supporting this role in other cancers such as hematopoietic malignancies^127^ and NSCLC.^128^ This overexpression is likely contributing to increased survival signaling during treatment.^9^ Cytotoxic drugs such as docetaxel induce significant PIM overexpression in RWPE-2 benign prostate epithelial cells at the protein (as much as 6.25-fold) and mRNA (2–4-fold) levels.^9^ This may be caused by a simultaneous increase in phospho-STAT3, which upregulates PIM.^9,129^

Knock-out studies in PCa cell models (PCA3 cell line) suggested that the absence of PIM increases apoptotic cell death, which becomes more prominent after exposure to chemotherapeutic anti-tubulin agents such as paclitaxel, vincristine, evodiamine or colchicine.^129^ However, this effect was not dependent on tubulin polymerization but rather on blocking DNA repair mechanisms via inhibition of DNA kinases such as ATM (ataxia-telangiectasia mutated kinase) or DNA-PK (DNA-dependent protein kinase) and upregulating H2A.X (H2A histone family member X) phosphorylation, which is involved in DNA fragmentation as a part of the apoptotic mechanism. Moreover, similar results regarding an increase in H2A.X phosphorylation and PARP-1 cleavage as well as reduced ATM and RPA32 (replication protein A 32 kDa subunit) phosphorylation were demonstrated when a PIM inhibitor, quercetin, was used together with paclitaxel in PIM-expressing cells.^129^

Further evidence that PIM1 overexpression protects tumor cells from chemotherapy-induced apoptosis is that its downregulation leads to increased sensitivity to treatment.^9^ This effect may be mediated by NF-κB signaling, particularly by the RELA (nuclear factor NF-kappa-B P65 subunit) component of the complex.^9^ Upregulation of NF-κB itself has been shown to weaken the pro-apoptotic effects of chemotherapeutic agents such as paclitaxel, likely by affecting MDR1 (multidrug resistance protein 1) and other anti-apoptotic genes.^130,131^ Moreover, as mentioned above, PIM1-L and its target BCRP are both overexpressed in mitoxantrone- and docetaxel-resistant PCa, and PIM knockdown has been shown to restore sensitivity to chemotherapy in affected cells.^104^

Another study on PIM inhibitors and chemotherapeutic agents in PCa suggests that a combined therapy involving paclitaxel and PIM inhibition has a synergistic effect on reducing cell viability, affecting cell cycle distribution and reducing apoptosis.^132^

## PIM and immunotherapy

Upstream molecules modifying the expression and function of PIM include interleukins such as IL-2 (interleukin-2), IL-3 (interleukin-3) and IL-6. As such, it has been suggested that PIM could be targeted indirectly using immunotherapeutics. In PCa cell models, an IL-6 antibody has been shown to decrease PIM1 expression.^15^ Moreover, PIM1-specific antibodies decreased prostate tumor and leukemia cell growth, increased apoptosis and affected levels of PIM1, HSP90, AKT and caspase pathway proteins.^133^ PIM kinases have been shown to phosphorylate glycogen synthase kinase 3β (GSK3B) and the tumor suppressor FOXP3 and block their influence on prostate cancer cells, leading to increased cell migration and adhesion.^134^ Moreover, FOXP3 has been shown to be involved in the development of T regulatory cells (T-regs),^135^ which have been implicated in driving tumor resistance to immunotherapeutics.^136,137^ Targeting T-regs with a PIM1 inhibitor helped to improve their suppressive activity.^135,136^ Kaempferol, a plant flavonoid with PIM1-inhibitory properties, has been shown to exhibit protective functions against high-dose IL-2-induced toxicity due to its positive effect on T-regs. Treatment with kaempferol led to lower body weight loss and higher survival than the control treatment.^138^ In Hodgkin lymphoma, PIM kinases have been shown to contribute to the immunosuppressive environment through modulation of PD-L1/2 (programmed death-ligand) activity, and although the PIMs are not key drivers of this phenotype, there is some rationale to support investigating the co-targeting of the PIM family (via the pan-PIM inhibitor SEL24) with PDL-1 (139). A recent study on adoptive T-cell therapy (ACT) has suggested that PIM inhibition could be used alongside anti-PD1 (programmed death receptor 1) therapy.^139^ The cells obtained from PIM knock-out mice had lower glycolytic activity, S6 phosphorylation, interferon gamma secretion, and ROS levels than those from wild-type mice, which translates to decreased T-cell death, an increased memory phenotype, and superior tumor control and mouse survival. A similar outcome was demonstrated following treatment with AZD1208. These effects were potentiated by the use of an anti-PD1 antibody (triple combination therapy), prolonging mouse survival, decreasing tumor growth and improving T-cell migration.^140^ Multiple studies have suggested the use of immunotherapeutics, perhaps alongside PIM inhibitors, to overcome the mechanisms of resistance to treatment and improve the functional outcomes.^27,133^

## Concluding remarks

Inhibition of PIM in PCa has attracted much attention as a potential monotherapeutic over the last decade, with growing in vitro, in vivo and early clinical trial data supporting this approach to varying degrees. However, despite extensive investigation, no PIM inhibitors have yet been approved for patient treatment in PCa. Targeting PIM alone has been suggested to be ineffective because of the existence of signaling bypass tracks that could lead to acquired resistance and because inhibitor toxicity limits the safe and tolerable dose. Utilizing a combined therapeutic approach, with both PIM inhibitors and other signal transduction inhibitors or other standard PCa treatments, could overcome these issues, reducing toxicity and improving patient quality of life and potentially survival. These benefits have already been demonstrated for some combinations in vitro and in vivo, and clinical trials are warranted to determine potential patient benefits. Following a demonstration of the efficacy of such strategies, it will be crucial to identify predictive companion biomarkers that can determine which patient groups are likely to benefit from each combination therapy and thus adopt a more personalized approach to treatment, matching the appropriate combination with the appropriate patient. This could be achieved by using proteomics^141^ or RNA sequencing^142^ in patients treated with the specific therapies. In summary, we believe that co-targeting PIM with rational treatment combinations is a promising concept for improved treatment of PCa.
